# Amide-Based Anti-Wear/Extreme-Pressure Additives for Silica-Thickened Greases: Structure and Wear Resistance

**DOI:** 10.3390/molecules30122492

**Published:** 2025-06-06

**Authors:** Jolanta Drabik, Kamil Korasiak, Justyna Chrobak, Rafał Kozdrach, Julia Woch, Michał Cyl, Magdalena Zarębska, Bernadetta Kaźmierczak, Jolanta Iłowska, Katarzyna Szymańska

**Affiliations:** 1 Łukasiewicz Research Network–Institute for Sustainable Technologies, 26-600 Radom, Poland; jolanta.drabik@itee.lukasiewicz.gov.pl (J.D.); rafal.kozdrach@itee.lukasiewicz.gov.pl (R.K.); bernadetta.kazmierczak@itee.lukasiewicz.gov.pl (B.K.); 2 Łukasiewicz Research Network–Institute of Heavy Organic Synthesis “Blachownia”, 47-225 Kędzierzyn-Koźle, Poland; justyna.chrobak@icso.lukasiewicz.gov.pl (J.C.); julia.woch@icso.lukasiewicz.gov.pl (J.W.); colocd.cyl@gmail.com (M.C.); magdalena.zarebska@icso.lukasiewicz.gov.pl (M.Z.); jolanta.ilowska@icso.lukasiewicz.gov.pl (J.I.); 3 Joint Doctoral School, Silesian University of Technology, Akademicka 2a, 44-100 Gliwice, Poland; 4 Department of Chemical Engineering and Process Design, Faculty of Chemistry, Silesian University of Technology, 44-100 Gliwice, Poland; katarzyna.szymanska@polsl.pl

**Keywords:** lubricants, greases, anti-wear properties, extreme-pressure properties, silica nanoparticles, SBA-15, amides

## Abstract

The lubricating properties of three secondary amides were evaluated using the four-ball apparatus method. It was found that the studied amides—N-propylpropanamide (AC3C3), N-propyloctanamide (AC3C8), and N-propyldodecanamide (AC3C12)—could be a promising group of new AW/EP (anti-wear/extreme-pressure) additives for lubricants, especially for silica-thickened greases. Of the amides tested, AC3C8 was found to have the best properties. The synthesized amide structures were immobilized on SBA-15 nanosilica and examined as described above. Notably, SBA-15 has not previously been reported as a potential lubricant additive. The results of the tribological tests showed that SBA-15-immobilized amides performed better than non-immobilized amides. Nevertheless, the lack of stability of the amide-grafted SBA-15 when dispersed in oil limits its use in targeted formulations and should be improved through further research. By testing a silica-thickened grease, a synergistic effect was observed between the free-amide AC3C8 and a commercially available additive containing butylated triphenyl phosphate. A 240% increase in the Goz40 parameter (anti-wear properties) and a 150% increase in welding load (extreme-pressure properties) were obtained with the addition of 2%*w*/*w* of commercial additive and 3%*w*/*w* of AC3C8 to a base oil.

## 1. Introduction

Lubricants play a vital role in ensuring the efficiency of modern industry, machinery, and transport. Due to increasing environmental regulations and restrictions, there is now a significant demand for highly stable, pure, and biodegradable base oils, particularly those based on polyalphaolefins (PAOs) [1], as well as a need for novel, environmentally friendly additives.

Modern lubricants are usually complex mixtures of lubricating oils and various additives, including anti-wear (AW) additives, extreme-pressure (EP) additives, pour point depressants, friction modifiers, emulsifiers (or demulsifiers), antioxidants, anti-corrosion agents, rheology modifiers, and many others [2]. Of these, additives providing anti-wear properties appear to be the most important. The reduction in wear not only limits the destruction of mechanical parts but also reduces maintenance costs and energy consumption, thus improving the cost efficiency of machinery. The boundary lubrication of triboparts (surfaces in relative motion) requires the formation of a protective layer (film) between the working parts. Therefore, chemical compounds capable of forming thin, protective films could be potential AW/EP additives. Typically, these compounds contain polar oxygen/nitrogen/sulfur/phosphorus groups, which enable the compound to adsorb onto or chemically react with metal surfaces [3]. They are known as organic friction modifiers (OFMs) and have a surfactant-like structure with polar groups and alkyl chains [4].

One of the oldest and most widely used groups of lubricant additives is zinc alkylditiophosphates (ZDDPs) [5,6], which is due to their AW/EP, anti-corrosion, and antioxidant properties [2]. However, recent studies are focused on replacing or reducing the use of ZDDPs due to their negative impact on human health and the environment [7]. ZDDPs release harmful zinc, sulfur, and phosphorus compounds into the environment. Moreover, the decomposition products of ZDDPs are poisonous to catalytic converters [7,8]. For these reasons, there is a noticeable trend toward the complete removal of ZDDPs from lubricants and a growing interest in new environmentally friendly additives.

Fatty acids and their derivatives, including amides, are widely considered to be metal-free, sulfur-free, and phosphorus-free OFMs due to their amphiphilic structure and proven ability to adsorb onto steel surfaces [9]. Due to the significant corrosiveness of free fatty acids [10], their potential applications are limited; however, fatty acid derivatives, such as amides, could make more promising additives. The AW and EP properties of primary amides (lauryl, mirystyl, palmityl, stearyl, oleyl, and erucic) were studied by Khalkar et al. [11]. The investigated amides provided up to a 35% reduction in wear scar diameter and an increase in the maximum welding load in several cases. The authors concluded that the tribological performance of primary amides increases with increasing the molecular weight. Although simple primary amides perform well as AW/EP additives, more sophisticated amide structures could provide additional features such as improved stability [12] or enable the formation of double-layered boundary films [13]. In recent years, a number of these amides have been characterized as follows: N-(2,2,6,6-tetramethyl-1-oxyl-4-piperidinyl)dodecaneamide [13,14], 1,3-dioleoamide-2-propyloleate (DOAPO) [12], and NIPAOA and NDEAOA (made by reacting oleamine with N-isopropylacrylamide and N,N-dimethylacrylamide, respectively) [15].

Our team has also conducted research [16] on environmentally friendly multifunctional additives based on amide/amino-based compounds, with a particular focus on quaternary ammonium salts containing an amide group. Even small amounts of these additives improved the anti-wear, antimicrobial, and antioxidative properties of the tested grease.

Another group of lubricant additives with a lot of potential are nanoparticles, such as graphene [17,18,19], molybdenum disulfide [20], tungsten disulfide [21], carbon nanotubes [22,23,24], silicon dioxide (silica), and copper nanoparticles [25]. The mechanisms by which nanoparticles improve the properties of lubricants are, as of yet, not well understood. The literature points to the following effects, which are observed in tribosystems when nanoparticles are used: the ball bearing effect [26], protective film formation [25], polishing [27], and mending [28]. Of all the types of nanoparticles mentioned above, silica nanoparticles are particularly promising due to their chemical and thermal stability, environmental inertness, and lack of health impacts. Although the long-term environmental impact of silica nanoparticles is still being discussed [29], various food and food-contact products containing silica nanoparticles are widely available [30,31]. The regulatory guidelines for silica are primarily focused on occupational exposure to respirable crystalline silica and have been introduced to mitigate the health risks associated with its use. The latest regulatory frameworks include the USA’s OSHA guideline from 2018, which sets the Permissible Exposure Limit at a 50 µg/m^3^ average over an 8-h workday, and the EU Directive 2017/2398 classifying respirable crystalline silica as a category 1B carcinogen. In 2024, the ECHA proposed stricter regulations on silicon dioxide and silicon compounds; however, these are still being considered for new applications and are expected to be introduced into industries such as cosmetics.

The possibility of using functionalized silica nanoparticles as lubricant additives has not been widely studied thus far, with only a few articles published on the use of different types of spherical silica nanoparticles as lubricant additives: raw (non-functionalized) [32]; amine-functionalized [33,34]; amide-functionalized [35,36,37]; (poly)lauryl methacrylate-functionalized [38]; amine-alkyl-bifunctionalized [39]; and ionic liquid-functionalized silica nanoparticles [40]. One of the most recent of these, that by Syed et al. [5], considered green silica nanoparticles combined with oleic acid as a promising additive, which enabled up to a 25% reduction in the amount of ZDDPs used in lubricants. Recent articles have considered silica nanoparticles as a potential additive in various groups of lubricants, including Pickering emulsions [41], semi-solid greases (where silica is used as a thickener) [42], and vegetable oils [43].

In this work, our two main objectives were (1) to determine the effect of the addition of secondary amides (N-propylpropanamide, N-propyloctanamide, and N-propyldodecanamide) on the properties of PAO-100-based lubricants and (2) to try to deposit these amides on silica carriers and use them as anti-wear additives. Secondary amides are known to be highly stable compounds [44,45] and, due to the presence of an additional alkyl chain, more similar to hydrocarbons than primary amides. Regulatory guidelines for the use of amides include the EPA’s evaluation of the toxicity of amides used in various applications and the EFSA’s evaluation of the safety of amide-containing substances used in food-related industries.

The examined lubricants were prepared using a group IV oil base and a certified thickener. According to the API’s oil classification, which divides base oils into five groups depending on their composition, physicochemical properties, and production technology, base oils meeting their ecological criteria belong to groups IV and V. According to the API classification, the use of group IV and V oils can contribute to the development of biodegradable lubricants that will not pose a threat to ecosystems.

**Figure 1:**
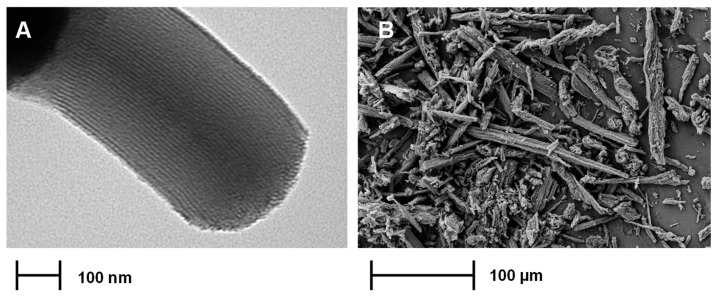
TEM (**A**) and SEM (**B**) images of SBA-15.

As previous research on silica nanoparticles as lubricant additives has mainly focused on spherical silica, we decided to investigate non-spherical silica nanoparticles in this work. We used SBA-15 (Santa Barbara No. 15) silica, modified with secondary amide compounds, as an additive with AW/EP properties. SBA-15 is a mesoporous silica with ordered internal channels (Figure 1A). The SBA-15 particle itself is spindle-shaped, with the size of its longitudinal dimension significantly exceeding that of its transverse dimension (Figure 1B). It has been successfully used as an adsorbent and catalyst support [46,47,48,49,50,51,52], but not as a potential lubricant additive. Other similarly shaped, elongated nanomaterials, such as carbon nanotubes, have been successfully used in lubricants, resulting in improved tribological performance [23,24,53,54,55,56]. The mechanisms behind this improvement were reported to have a rolling effect and mending effect [57].

To summarize, there is a growing demand for new, environmentally friendly additives for lubricants. Carboxylic acid and its derivatives, particularly amides, could be considered green additives, while for environmental reasons, nanosilica could be particularly interesting. Recent approaches using spherical silica nanoparticles could be extended to consider other morphologies, including elongated “rod-like” silicas such as SBA-15. In addition, combining the chemical properties of amides with the beneficial carrier morphology of silica through covalent grafting may result in further tribological performance improvements for lubricants.

## 2. Results and Discussion

### 2.1. Characterization of Free Amides and SBA-15-Grafted Compounds

**Figure 2:**
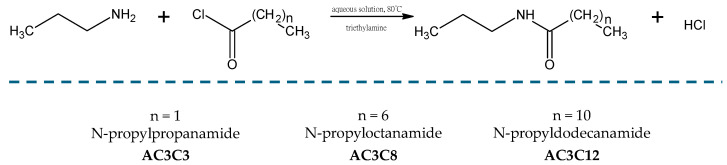
Scheme of the syntheses of the amide compounds.

In the first stage of this research, three amide compounds were synthesized: N-propylpropanamide (AC3C3), N-propyloctanamide (AC3C8), and N-propyldodecanamide (AC3C12). The scheme of their synthesis is shown in Figure 2, and the procedures used are described in detail in Section 3.2. According to the Reaxys database, AC3C3, AC3C8, and AC3C12 compounds have not previously been studied as potential lubricant additives.

**Figure 3:**
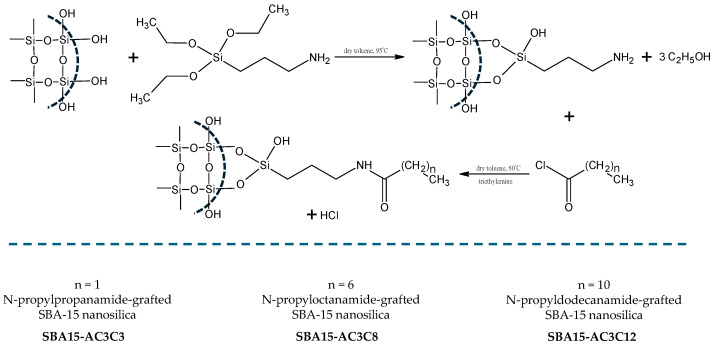
Scheme of the grafting of the amide compounds to SBA-15 silica nanoparticles.

The next step was to introduce the amide structures to the silica’s surface using the scheme shown in Figure 3. The procedures used are described in detail in Section 3.2. The obtained structures were labeled as follows: SBA15-AC3C3, SBA15-AC3C8, and SBA15-AC3C12.

**Figure 4:**
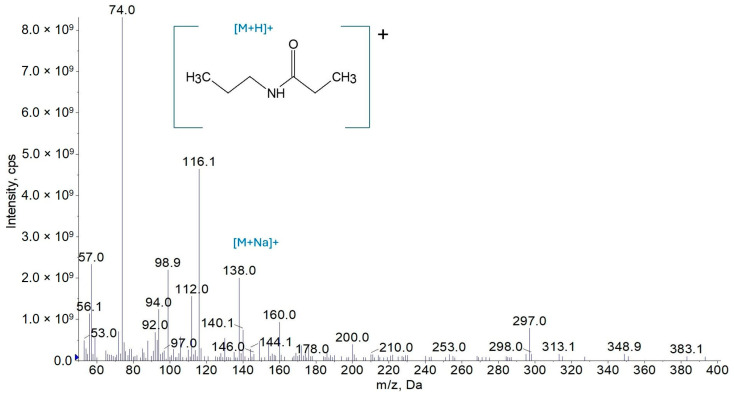
Mass spectrum of the AC3C3 amide in the Q3 scan mode.

**Figure 5:**
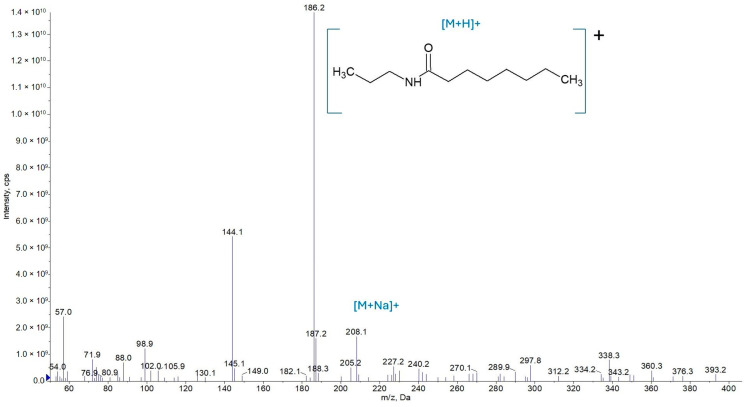
Mass spectrum of the AC3C8 amide in the Q3 scan mode.

**Figure 6:**
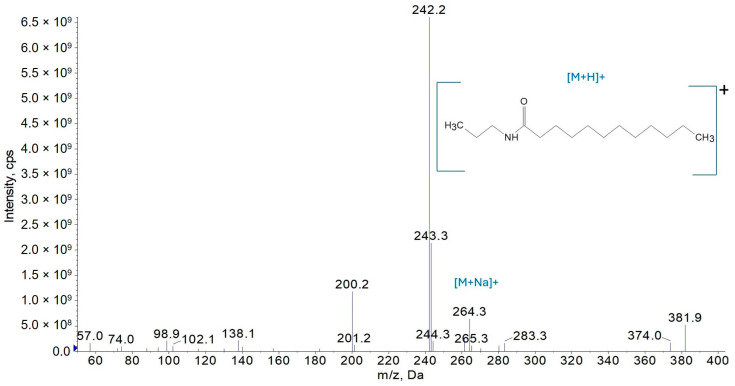
Mass spectrum of the AC3C12 amide in the Q3 scan mode.

The amides’ structures were confirmed via mass spectrometry. The protonated ions present in the sample, as detected in the third-quadrupole (Q3) scan mode, are shown in Figure 4, Figure 5 and Figure 6 for amides AC3C3, AC3C8, and AC3C12, respectively.

Based on this analysis, peaks were observed in AC3C3 (Figure 4) at *m*/*z* 116, corresponding to the molecular ion of the compound, and *m*/*z* 138, corresponding to its sodium adduct. Additionally, a well-ionizing peak at *m*/*z* 74, corresponding to the fragment [M − C_3_H_6_]^+^, was recorded.

A detailed description of the proposed fragmentation pathways is provided in the Supplementary Materials (Figures S1–S3).

Peaks were observed in AC3C8 (Figure 5) at *m*/*z* 186 Da [M + H]^+^, corresponding to the protonated pseudomolecular ion of the amide, 208 Da [M + Na]^+^, corresponding to its sodium adduct ion, and 144 Da, which is the fragmentation product of the 186 Da ion [M − C_3_H_6_]^+^.

In the Q3 spectrum of AC3C12 (Figure 6), the most intense peaks were identified as being at *m*/*z* 242 (molecular ion of the amide) and *m*/*z* 264 (sodium adduct). Similarly to previous observations, a fragment peak resulting from the loss of the C_3_H_6_ group was also recorded at *m*/*z* 200.

The interpretation of the spectra of AC3C3, AC3C8, and AC3C12 enables the identification of their structures, which enables us to confirm the presence of an amide bond and a long aliphatic chain.

The presence of amide compounds in the functionalized SBA-15 silica samples was confirmed via infrared spectroscopy.

**Figure 7:**
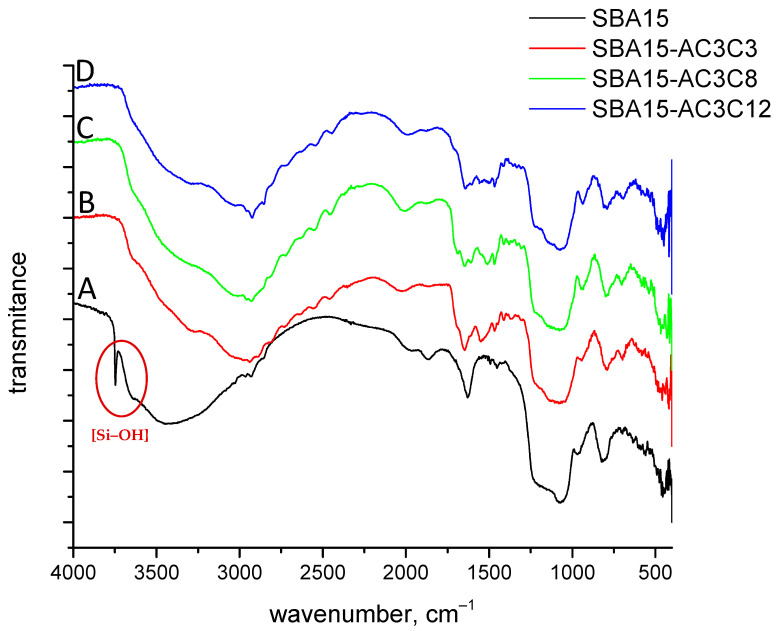
FT-IR spectra of raw and amide-grafted SBA-15 nanosilica samples.

The wide band at 1000–1300 cm^−1^ is assigned to the asymmetric stretching vibrations of Si–O–Si bonds [58], while the band at 780–800 cm^−1^ is assigned to symmetric stretching vibrations [59] (Figure 7). The hydroxyl groups were observed as a wide band at 3000–3700 cm^−1^, and the narrow band at 3740 cm^−1^ corresponds to the isolated silanol groups Si–OH [58] (Figure 7A). After grafting the amide structures onto the silica, the 3740 cm^−1^ band was significantly reduced (Figure 7B–D), which proves the presence of covalent bonding between the silica and organic structures. The presence of amide structures was observed through the band at 2850–2950 cm^−1^, which is assigned to methylene group stretching vibrations in the alkyl chain [60,61], and the band at 1640–1660 cm^−1^, which is assigned to vibrations of the –CO–NH– bond [62].

### 2.2. Characterization of Mixtures of PAO Base Oil and Investigated Additives

Subsequently, after the syntheses, the samples containing the base oil and either the obtained free amides or amide-grafted SBA-15 were prepared and examined.

The preparation procedures are described in Section 3.3. The wear resistance properties were measured via tribological tests conducted using a four-ball apparatus, as described in Section 3.1.3. Wear resistance properties include the limiting load of wear Goz, which is a unit load that determines the pressure in the friction node at a given constant load and is calculated on the basis of the average diameter of the scars (d) formed on stationary balls during the running of an apparatus under this load. The results are shown in Table 1.

**Figure 8:**
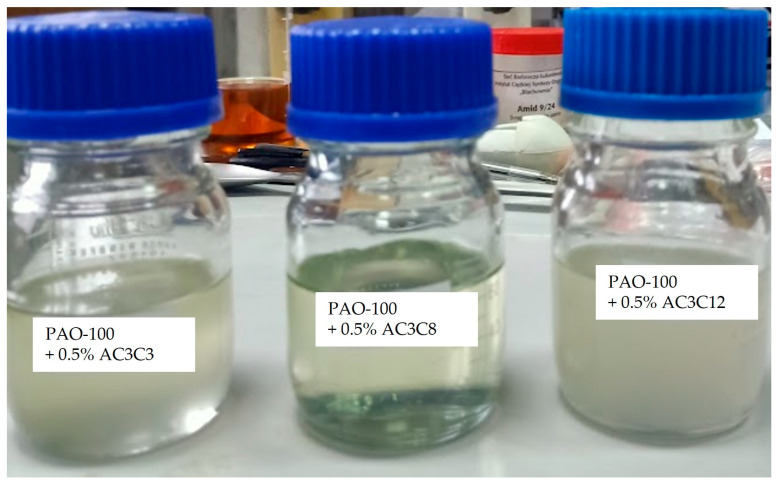
Visual appearance of PAO-100 oil containing the investigated amides.

The results (Table 1, rows 1–4) show that the addition of a small amount (0.5%*w*/*w*) of free amides improves the anti-wear properties of the base oil, as evidenced by the decrease in wear diameter and increase in G_OZ40_. The smallest changes were observed for AC3C3 (Table 1, row 2) and the largest for AC3C8, where the G_OZ40_ was doubled. Furthermore, only this amide (AC3C8) retains its transparency in PAO-100 (Figure 8).

As the next step, 0.5%*w*/*w* of pure SBA-15 (Table 1, row 5) or SBA-15 modified with suitable amides (Table 1, rows 6–8) was added to the PAO-100. The addition of even small amounts of unmodified SBA-15 resulted in a reduction in the anti-wear properties of the lubricant. However, the addition of amide-modified forms of the silica resulted in the oil having significantly improved anti-wear properties. Moreover, these parameters were even better than those of the lubricants containing free amides. For example, for PAO-100 containing AC3C8, the wear scar diameter was 0.34 mm (Table 1, row 3), while it was 0.22 mm when the same amide was deposited on SBA-15 (Table 1, row 7); i.e., we obtained a 35% reduction in the diameter of wear scars compared to PAO-100 containing free amides and more than a 50% reduction compared to PAO-100 oil alone (Table 1, row 1). For comparison, 0.5%*w*/*w* commercial spherical silica modified with AC3C8 amide was added to the PAO-100 base oil (Table 1, row 9). An improvement in its anti-wear properties was observed, but the results were not as substantial as those seen with SBA-15-AC3C8 (Table 1, row 7). Spherical nanosilica grafted with AC3C12 was also investigated by López et al. [35]. In their work, AC3C12-grafted spherical nanosilica provided a friction coefficient reduction of approx. 40%, but the wear scar diameter was significantly increased. The comparison between the properties of SBA-15-AC3C12 presented in this work and those of the (spherical) silica-AC3C12 reported by López et al. indicates that nanosilica morphology potentially plays a key role in the tribological performance of these lubricants.

Unfortunately, sedimentation during storage was observed in all samples containing added silica, indicating the need for further research to improve the stability of their composition. Similar stability issues were reported by López et al. [35]. A potential solution could be introducing nanosilica into the oil system without drying it, as has been reported by Sui et al. [63]; this will be the focus of our future research.

### 2.3. Analysis of Silica-Thickened Greases Containing Free Amides

Since the amide-modified SBA-15 silica formed precipitates in the PAO-100 oil, free amides were chosen for the practical tests. PAO-100 oil doped with 8%*w*/*w* Aerosil^®^-fumed silica (Evonik) was used as the base grease. AC3C8 was then added, as it led to the best anti-wear properties (Table 1, row 3). The effect of the amount of amide added on the anti-wear factors was determined. The preparation of these samples is described in Section 3.4. Their anti-wear and extreme-pressure properties were measured via tribological tests conducted with a four-ball apparatus, as described in Section 3.1.3. The wear resistance properties measured include the limiting load of wear, Goz, and the welding load—the lowest applied load at which there is a significant increase in frictional resistance, indicating a break in the lubricating film, i.e., welding of the rotating ball to the three stationary balls. The results are shown in Table 2.

When the AC3C8 amide was added to the base grease at 1, 3, and 5%*w*/*w* (Table 2, rows 2–4), the tests showed that it was the 3%*w*/*w* addition that reduced the wear diameter from 0.59 mm (for the base grease, Table 2, row 1) to 0.46 mm (Table 2, row 3). For comparison, a commercial additive was added to the base grease at the manufacturer’s recommended level of 2%*w*/*w* (Table 2, row 5). The commercial additive reduced the wear diameter compared to the base grease, but its results were significantly worse than the base grease combined with 3%*w*/*w* of our AC3C8 additive.

Unexpectedly, the addition of both the commercial additive at 2%*w*/*w* and AC3C8 at 3%*w*/*w* led to the best anti-wear parameters (Table 2, row 6): a wear diameter of 0.32 mm, Goz_40_ of 2000 N·mm^−2^ (240% increase), and a welding load of 7848 N (150% increase). We assume that there is synergy between butylated triphenyl phosphate and AC3C8. Synergy (or antagonism) between various additives used in lubricants has been previously reported [64,65,66,67,68,69,70]. We intend to investigate this phenomenon in future research.

## 3. Materials and Methods

### 3.1. Analytical Methods

#### 3.1.1. Mass Spectrometry

The 4500 Q-TRAP mass spectrometer (AB Sciex, Framingham, MA, USA), featuring an electrospray ionization (ESI) source and a triple quadrupole-ion trap analyzer managed by the Analyst 1.7.2 software, was used for the identification and structural analysis of the compounds in the samples. The ESI source was operated in the positive-ion mode under the following parameters: a curtain gas pressure of 20 psi, a nebulizer gas pressure of 10 psi, and a source voltage set to 4500 V. Nitrogen was employed as the curtain gas and collision gas.

The samples were analyzed in the Q3 scan mode, during which the first quadrupole and collision cell were disabled, allowing all ions to be scanned at the third quadrupole. An EPI scan with a collision energy ramp was utilized to confirm the structure of the detected compounds.

Approximately 10 mg of each sample was weighed and dissolved in 10 mL of LC-MS-grade methanol before being diluted 1000-fold. The prepared solutions were introduced into the mass spectrometer via direct injection using a syringe pump at a flow rate of 10 μL/min, with the ions scanned across an *m*/*z* range of 50 to 1000 Da. For clarity, the displayed mass spectra were limited to a narrower *m*/*z* range. The identification of the selected compounds was based on their molecular masses and characteristic fragment ion patterns, with their structures further confirmed through a fragmentation analysis.

#### 3.1.2. FT-IR Spectroscopy

FT-IR spectroscopy was carried out using a Nicolet™ iS50 FT-IR spectrometer from Thermo Fischer Scientific (Waltham, MA, USA). Prior to the analyses, KBr was ground in an agate mortar and dried at 120 °C (for a minimum of 24 h). The samples (5%*w*/*w*) were then mixed with the previously prepared KBr and dried again at 100 °C. The analysis was then performed within the wavelength range of 400–4000 cm^−1^.

#### 3.1.3. Tribological Tests

Tribological tests were carried out using a T-02 four-ball apparatus produced by the Łukasiewicz Research Network–Institute for Sustainable Technologies in Radom and according to standardized requirements.

The anti-wear properties were assessed according to the WTWT-94/MPS-025 Military Temporary Technical Requirements standard for testing the anti-wear properties of propellant and lubricant materials [71]. Tests were carried out three times under the following conditions: an applied load of 392.4 N (40 kgf); a temperature of 20 °C; a spindle speed of 500 rpm; and a time of 3600 s. The wear scars were measured to 0.001 mm accuracy using a microscope; the results were rounded to the nearest hundredth of a millimeter according to the standard. A statistical analysis (ANOVA) of the obtained wear scar diameters was performed using the OriginPro 8.5.1. software. To prove that the wear scar diameters were significantly reduced, Tukey’s test for mean comparisons was performed (alpha = 0.05). The Goz_40_ (limiting load of wear at an applied load of 40 kgf) parameter was calculated according to the standard using the following formula:

$${Goz}_{40}=0.52\times \frac{392.4}{d^{2}}$$

where 392.4 is the load of the friction node in [N], and d is the average diameter of the scars in [mm] formed on the three immobilized steel bearing balls during the test.

The extreme-pressure properties (welding load) were assessed according to the PN-C-04147:1976 standard for testing the lubrication properties of lubricating oils and greases [72]. The applied load was increased gradually until the steel bearing balls started to become welded together. The tests were repeated to confirm the results.

### 3.2. Synthesis Procedures

#### 3.2.1. Synthesis of N-Propylpropanamide (AC3C3)

In a three-neck flask equipped with a condenser, a dropping funnel, a thermometer, and a dipole stirrer, 0.8 mol of propylamine (Sigma Aldrich, St. Louis, MO, USA, 99%), 0.8 mol of triethylamine (Avantor/POCh pure p.a., Gliwice, Poland), and 350 mL of distilled water were rapidly mixed under nitrogen at ambient temperature. After increasing the temperature to 40 °C, 0.8 mol of propionyl chloride (Sigma Aldrich, 98%) was added dropwise. After this addition, the mixture was stirred for 180 min, at which point it had a homogenous, slightly yellow appearance. The reaction mixture was then treated with a (2:1 vol.) mixture of n-hexane (Chemland, pure p.a., Stargard, Poland) and chloroform (Avantor/POCh, pure p.a., Gliwice, Poland) and mixed vigorously for several minutes. The two-phase mixture was separated, and the upper (organic) layer was washed with distilled water three times. Next, chloroform and hexane were evaporated from the product using a rotary evaporator. A slightly yellow liquid product was obtained with a 24% yield. This relatively low yield was due to the several-step extraction process.

#### 3.2.2. Synthesis of N-Propyloctanamide (AC3C8)

In a three-neck flask equipped with a condenser, a dropping funnel, a thermometer, and a dipole stirrer, 0.8 mol of propylamine (Sigma Aldrich, 99%), 0.8 mol of triethylamine (Avantor/POCh pure p.a.), and 350 mL of distilled water were rapidly mixed under nitrogen at ambient temperature. After increasing the temperature to 40 °C, 0.8 mol of octanoyl chloride (Sigma Aldrich, 99%) was added dropwise. After this addition, the reaction mixture was stirred for 180 min. The resulting two-phase reaction mixture was separated, and the upper (organic) layer was washed with distilled water three times. A slightly yellow liquid product was obtained with an 84% yield.

#### 3.2.3. Synthesis of N-Propyldodecanamide (AC3C12)

In a three-neck flask equipped with a condenser, a dropping funnel, a thermometer, and a dipole stirrer, 0.6 mol of propylamine (Sigma Aldrich, 99%), 0.6 mol of triethylamine (Avantor/POCh pure p.a.), and 350 mL of distilled water were rapidly mixed under nitrogen at ambient temperature. After increasing the temperature to 40 °C, 0.6 mol of dodecanoyl chloride (Sigma Aldrich, 97%) was added dropwise, and the solution was then stirred for 180 min. After 1 h, the temperature was increased to 60 °C due to the significant increase in the viscosity of the reaction mixture. The two-phase reaction mixture was separated, and the upper (organic) layer was washed with hot distilled water three times. A white solid product was obtained with a 69% yield.

#### 3.2.4. Synthesis of SBA-15 Nanosilica

The conditions under which SBA-15 was synthesized were based on published methods [73]. In a closed beaker equipped with a dipole stirrer, 4.0 g of Pluronic P123 (Merck, Darmstadt, Germany) was dissolved in 120 mL of 2M HCl (Avantor/POCh) and 30 mL of distilled water. The mixture was stirred vigorously at 35 °C. After the Pluronic P123 was completely dissolved, 8,46 g of tetraethoxysilane (Sigma Aldrich) was added dropwise, and the mixture was then stirred for 20 h at 35 °C. Afterwards, the mixture was moved to a sealed container and kept at 80 °C for 24 h. The obtained silica was separated from the suspension using a Büchner funnel and vacuum. It was dried in ambient conditions for 4 days and then calcinated (500 °C) for 6 h.

#### 3.2.5. Synthesis of N-Propylpropanamide-Grafted SBA-15 Nanosilica (SBA15-AC3C3)

The synthesis conditions were based on published methods [35].

Step 1: In a three-neck flask equipped with a condenser, a dropping funnel, a thermometer, and a dipole stirrer, dry SBA-15 nanosilica (preparation procedure in Section 3.2.4) and 450 mL of toluene (Avantor/Stanlab, dried over molecular sieves and filtrated) were rapidly mixed under nitrogen at ambient temperature. Then, after increasing the temperature to 95 °C, (3-aminopropyl)triethoxysilane (Sigma Aldrich, 99%) was added dropwise. The molar ratio of silane coupling agent to nanosilica was 10 mmol/g. After the addition of (3-aminopropyl)triethoxysilane, the reaction mixture was stirred for 18 h. Toluene was evaporated from the mixture, and the solid remains were moved into a Soxhlet extractor, extracted using dichloromethane for 5 h, and then dried using a rotary evaporator.

Step 2: In a three-neck flask equipped with a condenser, a dropping funnel, a thermometer, and a dipole stirrer, aminated SBA-15 nanosilica and 450 mL of toluene (Avantor/Stanlab, dried over molecular sieves and filtrated) were rapidly mixed under nitrogen at ambient temperature. After increasing the temperature to 80 °C, propionyl chloride (Sigma Aldrich, 98%) was added dropwise, and the reaction mixture was then stirred for 18 h. The molar ratio of acyl chloride to aminated nanosilica was 10 mmol/g. Toluene was evaporated from the reaction mixture, and the solid remains were moved into the Soxhlet extractor, extracted using dichloromethane for 5 h, and then dried using a rotary evaporator.

#### 3.2.6. Synthesis of N-Propyloctanamide-Grafted SBA-15 Nanosilica (SBA15-AC3C8)

Step 1 proceeded the same as that in Section 3.2.5. Step 2 proceeded the same as that in Section 3.2.5 while using octanoyl chloride (Sigma Aldrich, 99%) instead of acyl chloride.

This synthesis was repeated using commercially available spherical silica nanoparticles (5–20 nm, Sigma Aldrich) for comparison purposes (Table 1, last row).

#### 3.2.7. Synthesis of N-Propyldodecanamide-Grafted SBA-15 Nanosilica (SBA15-AC3C12)

Step 1 proceeded the same as that in Section 3.2.5 Step 2 proceeded the same as that in Section 3.2.5 while using dodecanoyl chloride (Sigma Aldrich, 97%) instead of acyl chloride.

### 3.3. Preparation of Mixtures of PAO Base Oil and Investigated Additives

The PAO base oil (Spectrasyn™ 100, Exxon, Houston, TX, USA) was mixed with the investigated additives in a T-25 homogenizer (IKA) with a speed of 13,500 rpm for 20 min. The samples containing amide-grafted silica were additionally sonicated using a sonotrode (VC 505, Sonics, Newtown, CT, USA) with a 20 kHz frequency at 50% amplitude for 15 min.

### 3.4. Preparation of Greases

The PAO base oil (Spectrasyn™ 100, Exxon) was mixed with the investigated additives for 20 min using a mechanical stirrer (500 rpm). Aerosil^®^-fumed silica (thickener) (Evonik, Hanau, Germany) was added gradually during the homogenization process (which took place at 22,000 rpm in the T-25 homogenizer).

## 4. Conclusions

Our research has shown that amide compounds may be a promising group of new additives that improve the performance of lubricants. Our conclusions are as follows:

- AC3C8 provided the best wear protection, reducing the diameter of wear scars by approximately 25%.
- Grafting the amide compound onto SBA-15 silica nanoparticles improved the anti-wear properties of the lubricant by approximately a further 30%. To our knowledge, this is the first publication to use SBA-15 as a lubricant additive.
- SBA-15 grafted with AC3C8 amide resulted in better anti-wear properties than similarly modified spherical commercial silica. Unfortunately, both commercial silica and SBA-15 led to sedimentation during their storage in PAO-100 oil. This aspect of their application still needs to be improved. Stabilizing the dispersion of functionalized silica may depend on various factors, including the homogenization technique used and the conditions it was carried out under, the chemical structure of the ligands, and even the grafting conditions [63].
- Due to the sedimentation of the silica, free-amide AC3C8 was used for the real tests, which showed that using it at 3%*w*/*w* generated the best anti-wear properties. We have shown that the properties of this sample are better than those obtained with the commercial additive (Additive 1). The developed additive can be used wherever certified lubricants are required, such as in the food production industry. In this industry, a health quality certification is required for the finished lubricant when used in both food and non-food areas.
- AC3C8 showed a synergistic effect with the commercial additive, which contained butylated triphenyl phosphate as an anti-wear agent. The mechanism behind this synergy is unknown and should be studied further using SEM/EDS. The aim of these analyses will be the evaluation of the tribofilm created and include an elemental analysis.
- We assume that the anti-wear and extreme-pressure performance of AC3C8 depends on its polar amide bond and, therefore, its ability to adsorb onto the triboparts under boundary conditions, while the length of its carbon chain (C8) makes it properly soluble in PAO oil.
- The mechanism behind the performance improvement of the lubricant containing AC3C8 grafted onto SBA-15 nanosilica is probably related to its morphology. Similarly shaped, elongated nanoparticles, such as carbon nanotubes, have been successfully used in lubricants, resulting in improved anti-wear performance. Previous studies suggest that these carbon nanotubes are able to behave as (nano)rolling bearings, as well as a mending agent, filling in the cracks in the triboparts [57].

## 5. Patents

Patent application no. P.448060 (WIPO ST 10/C PL448060) entitled “Dyspersja oleju smarowego z dodatkiem na bazie nanocząstek krzemionki oraz sposób wytwarzania dyspersji oleju smarowego z dodatkiem na bazie nanocząstek krzemionki”.

Patent application no. P.451397 (WIPO ST 10/C PL451397) entitled “Smar plastyczny zagęszczony nanocząstkami krzemionki z dodatkiem amidu i sposób wytworzenia smaru plastycznego zagęszczonego nanocząstkami krzemionki z dodatkiem amidu”.

## Figures and Tables

**Table 1:** Wear resistance and visual appearance of PAO base oil containing the investigated amide-based additives.

Line	Model Dispersion	Wear Diameter, d, mm Mean ± SD	Significance of Wear Diameter Reduction, (T)rue/(F)alse *	Goz_40_, N·mm^−2^ Mean ± SD	Visual Appearance
1	PAO-100	0.46 ± 0.01	reference	950 ± 20	transparent liquid
2	PAO-100 + 0.5%*w*/*w* AC3C3	0.44 ± 0.03	F	1100 ± 200	cloudy liquid
3	PAO-100 + 0.5%*w*/*w* AC3C8	0.34 ± 0.02	T	1800 ± 200	transparent liquid
4	PAO-100 + 0.5%*w*/*w* AC3C12	0.36 ± 0.03	T	1500 ± 200	cloudy liquid
5	PAO-100 + 0.5%*w*/*w* SBA15	0.52 ± 0.02	T	750 ± 50	sedimentation observed
6	PAO-100 + 0.5%*w*/*w* SBA15-AC3C3	0.36 ± 0.02	T	1600 ± 100	sedimentation observed
7	PAO-100 + 0.5%*w*/*w* SBA15-AC3C8	0.22 ± 0.01	T	4200 ± 300	sedimentation observed
8	PAO-100 + 0.5%*w*/*w* SBA15-AC3C12	0.28 ± 0.01	T	2600 ± 200	sedimentation observed
9	PAO-100 + 0.5%*w*/*w* (spherical) silica **-AC3C8	0.35 ± 0.02	T	1700 ± 200	sedimentation observed

* Results from ANOVA analysis (Tukey’s test was used for mean comparisons). The output table is included in the Supplementary Materials (Table S2). ** Silicon dioxide nanopowder (spherical, porous) had a 5–20 nm particle size (TEM). Sigma Aldrich (St. Louis, MO, USA), article no. 637246.

**Table 2:** Wear resistance and welding load of greases containing the investigated amide-based additives.

Line	Grease	Wear Diameter, d, mm Mean ± SD	Significance of Wear Diameter Reduction, (T)rue/(F)alse *	Goz_40_, N·mm^−2^ Mean ± SD	Welding Load, N
1	PAO-100 + Aerosil^®^ (base grease)	0.59 ± 0.01	reference	590 ± 20	3090.1
2	base grease + 1.0%*w*/*w* AC3C8	0.55 ± 0.01	T	680 ± 20	3090.1
3	base grease + 3.0%*w*/*w* AC3C8	0.46 ± 0.01	T	960 ± 40	3924.0
4	base grease + 5.0%*w*/*w* AC3C8	0.61 ± 0.01	F	550 ± 20	3924.0
5	base grease + 2.0%*w*/*w* Additive-1 **	0.55 ± 0.01	T	670 ± 20	3924.0
6	base grease + 2.0%*w*/*w* Additive-1 ** + 3.0%*w*/*w* AC3C8	0.32 ± 0.01	T	2000 ± 100	7848.0

* Results from ANOVA analysis (Tukey’s test used for mean comparisons). The output table is in the Supplementary Materials (Table S3). ** Additive-1—a commercially available additive containing butylated triphenyl phosphate as an anti-wear agent.

## Data Availability

The data presented in this study are available upon request from the corresponding author.

## Supplementary Materials

The following supporting information can be downloaded at: https://www.mdpi.com/article/10.3390/molecules30122492/s1, Figure S1. Mass spectrum and assigned fragments of the AC3C3 amide in EPI scan mode. Figure S2. Mass spectrum and assigned fragments of the AC3C8 amide in EPI scan mode. Figure S3. Mass spectrum and assigned fragments of the AC3C12 amide in EPI scan mode. Table S1. Amide-grafted silica samples—FTIR peak assignment. Table S2. Oil samples. Wear diameters mean values comparison—Tukey test output. Table S3. Grease samples. Wear diameters mean values comparison—Tukey test output.
