# Supplementary material for: Amide-Based Anti-Wear/Extreme-Pressure Additives for Silica-Thickened Greases: Structure and Wear Resistance
Source: Molecules. 2025 Jun 6;30(12):2492. doi: 10.3390/molecules30122492 (PMC12195770; doi:10.3390/molecules30122492)
Supplement: Supplementary file 1 [file molecules-30-02492-s001.zip › molecules-3641864-supplementary.pdf]

Mass spectrometry

A molecular mass of 116 Da is detected as the primary molecular ion peak for the AC3C3 (Figure S1). The spectrum reveals a characteristic fragment at m/z 74, attributed to the loss of an alkyl group ($-C_3H_6$) from the molecular ion. A molecular mass of 186 Da is observed as the main molecular ion peak for the AC3C8 (Figure S2). The spectrum features characteristic fragments at m/z 144, 130, 102, and 88, resulting from sequential losses of alkyl groups ($-C_3H_6$, $-CH_2$,$-C_2H_4$, $-CH_2$, respectively) or larger structural fragments. A molecular mass of 242 Da is identified as the dominant molecular ion peak for the AC3C12 (Figure S3). The spectrum displays characteristic fragment peaks at m/z 200, 144, 130, and 88, arising from the stepwise loss of alkyl groups ($-C_3H_6$, $-C_4H_8$, $-CH_2$, and $-C_3H_6$, respectively) or larger structural units.

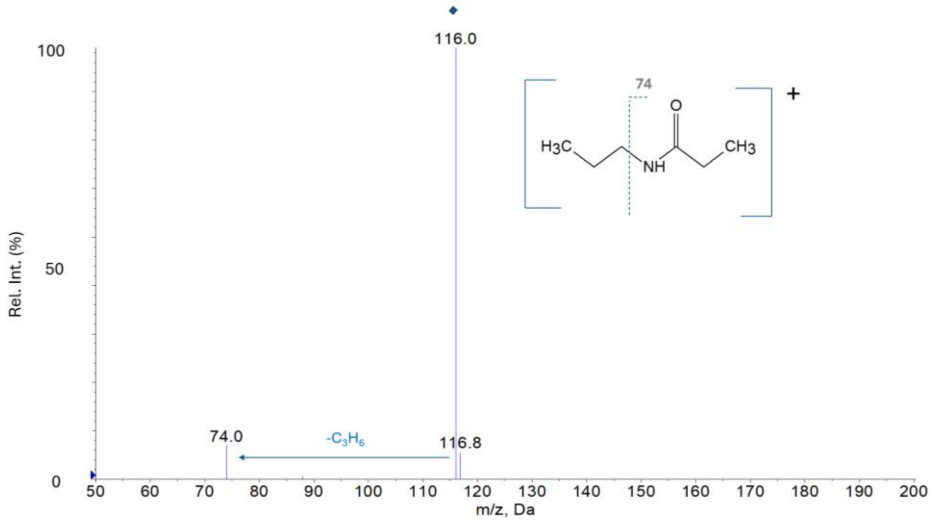

Figure S1. Mass spectrum and assigned fragments of the AC3C3 amide in EPI scan mode.

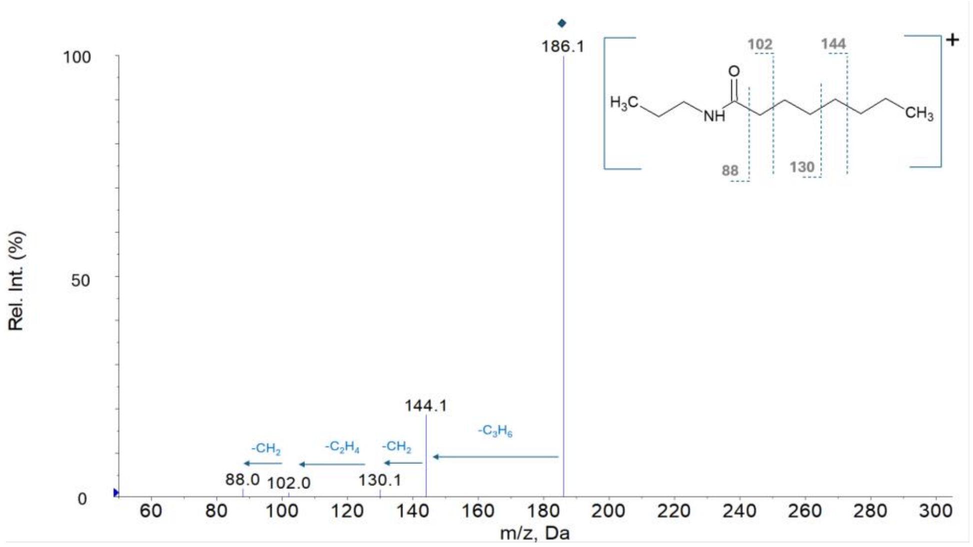

Figure S2. Mass spectrum and assigned fragments of the AC3C8 amide in EPI scan mode.

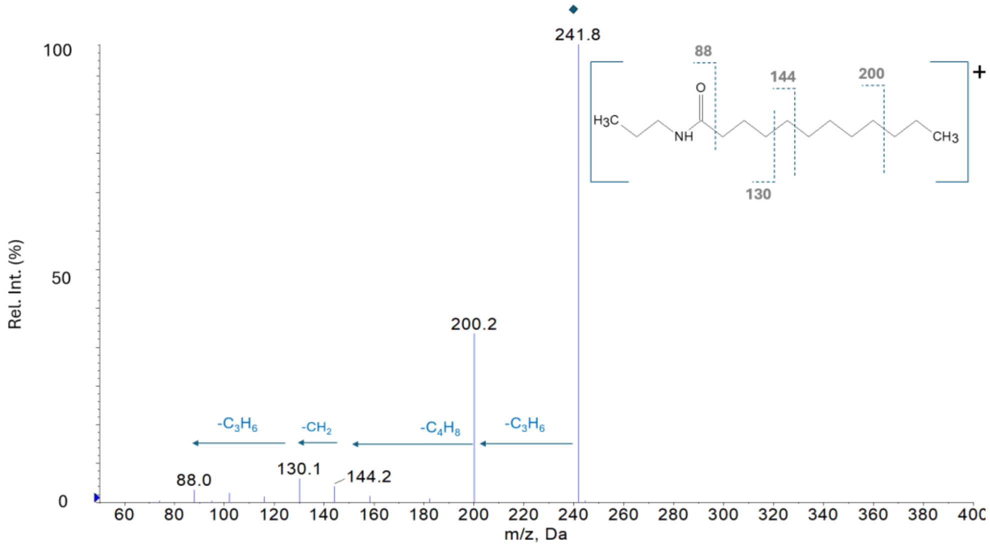

Figure S3. Mass spectrum and assigned fragments of the AC3C12 amide in EPI scan mode.

Amide-grafted silica samples – FTIR peak assignment

Table S1. Amide-grafted silica samples - FTIR peak assignment

Figure	Wavenumber, cm^{-1}	Vibrations type	Identified bond	Reference
7ABCD	1000–1300	asymmetric, stretching	Si–O–Si	[58]
7ABCD	780–800	symmetric, stretching	Si–O–Si	[59]
7ABCD	3000–3700	stretching	–OH	[59]
7A	3740	stretching	Si–OH	[58]
7BCD	2850–2950	stretching	$–CH_2–$	[60,61]
7BCD	1640–1660		–CO–NH–	[62]

Wear diameters – ANOVA analysis

Table S2. Oil samples. Wear diameters mean values comparison – Tukey test output.

Samples compared		Mean Diff	SEM	q value	Prob	Alpha	Sig	LCL	UCL
1.	2.								
PAO-100	PAO-100 + 0.5%w/w AC3C3	-0.03167	0.00963	4.65175	0.00847	0.05	1	-0.05702	-0.00631
PAO-100	PAO-100 + 0.5%w/w AC3C8	-0.13611	0.00963	19.99437	0	0.05	1	-0.16147	-0.11076
PAO-100	PAO-100 + 0.5%w/w AC3C12	-0.10444	0.00963	15.34262	9.09E-08	0.05	1	-0.1298	-0.07909
PAO-100	PAO-100 + 0.5%w/w SBA15	0.05	0.00507	13.941	0	0.05	1	0.03525	0.06475
PAO-100	PAO-100 + 0.5%w/w SBA15-AC3C3	-0.10611	0.00507	29.58589	7.41E-08	0.05	1	-0.12086	-0.09136
PAO-100	PAO-100 + 0.5%w/w SBA15-AC3C8	-0.25	0.00507	69.70499	0	0.05	1	-0.26475	-0.23525
PAO-100	PAO-100 + 0.5%w/w SBA15-AC3C12	-0.19	0.00507	52.97579	0	0.05	1	-0.20475	-0.17525
PAO-100	PAO-100 + 0.5%w/w (spherical)silica-AC3C8	-0.12	0.00567	29.9261	7.53E-08	0.05	1	-0.13649	-0.10351

Table S3. Grease samples. Wear diameters mean values comparison – Tukey test output.

Samples compared		Mean Diff	SEM	q value	Prob	Alpha	Sig	LCL	UCL
1.	2.								
base grease	base grease+ 1.0%w/w AC3C8	-0.04	0.01027	5.51024	0.00236	0.05	1	-0.06982	-0.01018
base grease	base grease+ 3.0%w/w AC3C8	-0.13	0.01027	17.90829	0	0.05	1	-0.15982	-0.10018
base grease	base grease+ 5.0%w/w AC3C8	0.02056	0.01027	2.83165	0.34828	0.05	0	-0.00926	0.05037
base grease	base grease+ 2.0%w/w Additive-1	-0.25278	0.01027	34.82168	2.64E-08	0.05	1	-0.2826	-0.22296
base grease	base grease+ 2.0%w/w Additive-1 + 3.0%w/w AC3C8	-0.03944	0.01027	5.43371	0.00284	0.05	1	-0.06926	-0.00963
